# Children With Metabolically Healthy Obesity: A Review

**DOI:** 10.3389/fendo.2019.00865

**Published:** 2019-12-10

**Authors:** Rade Vukovic, Tiago Jeronimo Dos Santos, Marina Ybarra, Muge Atar

**Affiliations:** ^1^Department of Pediatric Endocrinology, Mother and Child Healthcare Institute of Serbia “Dr Vukan Cupic”, Belgrade, Serbia; ^2^School of Medicine, University of Belgrade, Belgrade, Serbia; ^3^Department of Preventive Medicine and Public Health, Universidad Autónoma de Madrid, Madrid, Spain; ^4^Research Center of Sainte Justine University Hospital, Université de Montréal, Montreal, QC, Canada; ^5^Centre Armand-Frappier, Institut National de la Recherche Scientifique, Université du Québec, Laval, QC, Canada; ^6^Department of Pediatric Endocrinology, School of Medicine, Demirel University, Isparta, Turkey

**Keywords:** obesity, children, metabolically healthy obesity, metabolic syndrome, pediatric obesity

## Abstract

Children with “metabolically healthy obesity” (MHO) are a distinct subgroup of youth with obesity, who are less prone to the clustering of cardiometabolic risk factors. Although this phenotype, frequently defined by the absence of metabolic syndrome components or insulin resistance, was first described during the early 1980s, a consensus-based definition of pediatric MHO was introduced only recently, in 2018. The purpose of this review was to concisely summarize current knowledge regarding the MHO phenomenon in youth. The prevalence of MHO in children varies from 3 to 87%, depending on the definition used and the parameters evaluated, as well as the ethnicity and the pubertal status of the sample. The most consistent predictors of MHO in youth include younger age, lower body mass index, lower waist circumference, and lower body fat measurements. Various hypotheses have been proposed to elucidate the underlying factors maintaining the favorable MHO phenotype. While preserved insulin sensitivity and lack of inflammation were previously considered to be the main etiological factors, the most recent findings have implicated adipokine levels, the number of inflammatory immune cells in the adipose tissue, and the reduction of visceral adiposity due to adipose tissue expandability. Physical activity and genetic factors also contribute to the MHO phenotype. Obesity constitutes a continuum-increased risk for cardiometabolic complications, which is less evident in children with MHO. However, some findings have highlighted the emergence of hepatic steatosis, increased carotid intima-media thickness and inflammatory biomarkers in the MHO group compared to peers without obesity. Screening should be directed at those more likely to develop clustering of cardiometabolic risk factors. Lifestyle modifications should include behavioral changes focusing on sleep duration, screen time, diet, physical activity, and tobacco smoke exposure. Weight loss has also been associated with the improvement of insulin sensitivity and inflammation. Further investigative efforts are needed in order to elucidate the mechanisms which protect against the clustering of cardiometabolic risk factors in pediatric obesity, to provide more efficient, targeted treatment approaches for children with obesity, and to identify the protective factors preserving the MHO profile, avoiding the crossover of MHO to the phenotype with metabolically unhealthy obesity.

## Introduction

The global epidemic of childhood obesity, with the accompanying rise in the prevalence of endocrine, metabolic, and cardiovascular comorbidities in youth, represents one of the most important public health issues of the modern world (1–4). The earlier occurrence and increase in the prevalence of both pediatric obesity and metabolic syndrome (MS) leads to a potential decline in life expectancy, meaning that the youth of today could be the first generation to live shorter lives than their parents (2, 4–6).

In the context of the childhood obesity pandemic, a distinct subgroup of youth with obesity less prone to the development of metabolic disturbances, called “metabolically healthy obese” (MHO), has come into focus (7, 8). Despite having obesity, individuals with MHO display a “favorable” metabolic profile, with preserved insulin sensitivity, normal blood pressure and glucose regulation, normal lipids and liver enzymes, as well as a normal hormonal, inflammation, and immune profile (9–13). First described and investigated in the population of adults with obesity, the MHO phenomenon has also been extensively studied and confirmed in young people with obesity (4, 7, 13).

Although MHO status might not necessarily translate into lower mortality, and can crossover to “metabolically unhealthy obese” (MUO) phenotype during puberty, defining the MHO subpopulation within the youth with obesity is of high importance in order to elucidate the mechanisms protecting against the clustering of cardiometabolic risk factors, and for its clinical, preventive, and therapeutic decision-making implications (4, 7, 13–17). For example, the clear distinction between youth with MHO and MUO could prove useful in providing more efficient and targeted treatment approaches for both of these groups of children with obesity (4, 13, 18). The purpose of this review was to provide a concise summary of the current knowledge regarding MHO phenomenon in childhood and adolescence, including definition, prevalence, predictors, underlying mechanisms, treatment, outcomes, and implications for future research and clinical practice.

## MHO in Childhood: Criteria and Definitions Used

**Figure 1:**
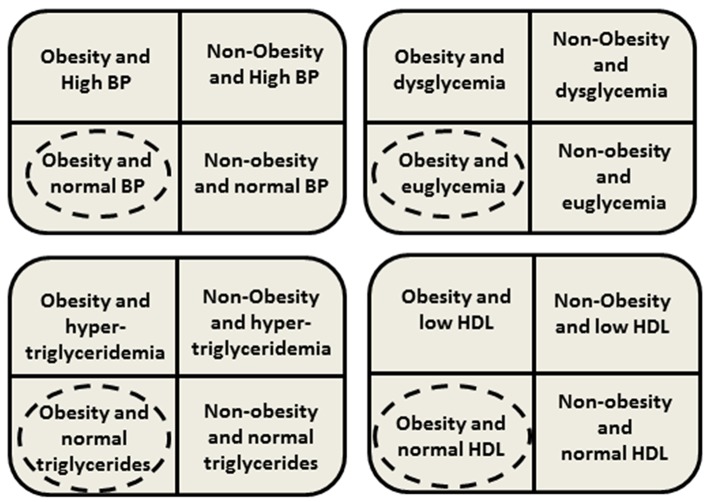
Presence or absence of clustering cardiometabolic risk factors. Dashed circles mean phenotype classified as metablically healthy obesity (MHO) in youth. BP, blood pressure; HDL, high density lipoprotein.

Although MHO phenotype was first described during the early 1980s, and is frequently defined (on the basis of known metabolic abnormalities associated with obesity) by the absence of MS components and/or preserved insulin sensitivity, a consensus-based definition of pediatric MHO was introduced only recently, in 2018 (4, 7, 13, 19, 20). Consequently, all the knowledge gained from the previous research is limited by the variety of definitions and criteria used for defining MHO. Despite the differences in the definitions used to identify MHO children, all available data undeniably confirms that a significant proportion of the youth with obesity displays a “favorable” metabolic phenotype [Figure 1; (4, 18)].

Until now, within the population of youth with obesity, children with MHO have been identified based either on the absence of MS components, preserved insulin sensitivity, or by various combinations of these criteria (8, 14, 16, 18, 21–24). Definitions have varied widely amongst different studies, even when defining obesity (or overweight) as *sine qua non* for youth with MHO. The cutoff values for obesity have differed amongst studies from the 85th to 97th percentile of body mass index (BMI) according to the Centers for Disease Control and Prevention (CDC) growth charts, while other researchers have used the World Health Organization (WHO) criteria, waist circumference (WC) or waist-to-height-ratio (4, 13). The definitions of the MHO phenotype in youth with obesity used in previous studies were most commonly based on: (a) absence of cardiometabolic risk factors (16, 25–27), (b) presence of ≤1 cardiometabolic risk factor (24, 27, 28), (c) presence of ≤2 cardiometabolic risk factors (29), (d) other criteria such as preserved insulin sensitivity or a combination of the above criteria (14, 30, 31). In all these studies, there has been great variation in the parameters considered as cardiometabolic risk factors [including high density lipoprotein (HDL), triglycerides, total cholesterol, low density lipoprotein (LDL), fasting plasma glucose, hemoglobin A1c (HbA1c), fasting plasma insulin, various indices of insulin sensitivity, etc.], with even more variation in the cutoff values defined using either absolute or percentile values, further contributing to the heterogeneity of the MHO definition (13). Furthermore, in addition to MS components or insulin sensitivity, it has been observed in several studies that individuals with MHO display other favorable phenotypic characteristics, such as the absence of hepatic steatosis, lower levels of liver enzymes, interleukin 6 and C-reactive protein compared with the rest of the population with obesity (22, 32–36).

Finally, in 2018, Damanhoury et al. proposed the first international consensus-based definition of MHO [Table 1; (13)]. The consensus was achieved to define obesity based on the BMI standard deviation score (BMI-SDS) > +2 SD using the WHO growth charts (13, 37). In respect of the definition, consensus was achieved that only children with obesity fulfilling all of the cardiometabolic criteria shown in Table 1 should be classified as with MHO (13). For the measure of glycemia criterion, no consensus was achieved, although the fasting plasma glucose with cutoff of ≤100 mg/dl (≤5.6 mmol/l), was the criterion for euglycemia most commonly used in previous studies of MHO in children (13). Hopefully, this definition could become a first step to standardization of the MHO phenotype in youth.

**Table 1:** Consensus-based definition of MHO in children (13).

**Only children with all of the following criteria fulfilled are classified as MHO**	
BMI-SDS	> +2 SD (using the WHO growth charts)
HDL	>40 mg/dl (>1.03 mmol/l)
Triglycerides	≤ 150 mg/dl (≤1.7 mmol/l)
Blood pressure (systolic and diastolic)	≤90th percentile
A measure of glycemia	Fasting plasma glucose ≤100 mg/dl (≤5.6 mmol/l) (the most commonly used euglycemia criterion)

*MHO, metabolically healthy obesity; BMI-SDS, body mass index standard deviation score; HDL, high density lipoprotein*.

One of the advantages of using the consensus-based definition proposed by Damanhoury et al. is the fact that most of the cutoff values are based on cutoffs provided by the International Diabetes Federation (IDF) definition of MS in youth, and are therefore widely used and known by clinicians and researchers dealing with pediatric obesity. The use of IDF MS cutoff values should facilitate comparison with future studies, and these fasting measures have also been shown to be reliable and valid for use in the population of youth with obesity (2, 13, 31). More importantly, this consensus-based definition is simple to use in the clinical setting, and is based on a Delphi process generated consensus of an international panel of 46 experts. This makes it more likely to be accepted by the majority of researchers in the field, which is probably the most important aspect in order to avoid further diversification in defining the MHO youth (13).

It should be noted, that the consensus-based MHO definition lacks important indicators of “metabolic health,” such as indicators of hepatic steatosis, immune, and inflammation markers. In addition, insulin resistance, a component considered to have a central role in the development of the obesity related cardiometabolic risk factors, is not included in this definition (2, 13). These shortcomings of the consensus definition should not be overlooked, although it would not be reasonable to expect availability of all these parameters in every future study of youth with MHO. One possible solution would be to use the consensus definition in all future studies of MHO in children as a starting point for a universal MHO definition. This approach would maximize the comparability of prevalence rates and clinical findings between studies, with the possibility of adding additional markers of “metabolic health” when feasible, while also presenting the findings based solely on the consensus definition of MHO.

The ideal definition of MHO should include (in addition to the MS components) the degree of visceral adiposity, insulin sensitivity, markers of hepatic steatosis, inflammatory, and immune parameters, although as yet, no consensus has been reached on the use of these parameters (or their cutoff values) in defining MHO (4). Thus, the inclusion of these parameters in the MHO definition at the present time would only aggravate the diversification in defining the MHO phenotype in children, leading both to difficulties in comparison of MHO prevalence rates, and to different findings between studies (31).

## Prevalence and Predictors of MHO in Childhood

The estimated prevalence of children with MHO has differed significantly in previous studies, in large part due to the various definitions used. As already stated, until 2018 there was no consensus regarding the definition of MHO in pediatrics and the criteria used for defining both the obesity and MHO/MUO status varied widely. Moreover, the groups studied were significantly distinct from one another (regarding gender, age, ethnicity, degree of obesity, etc.). Bearing all this in mind, the overall estimated prevalence of metabolically healthy phenotype in children of all weight status varied from 7 to 21%, whereas the prevalence of MHO among overweight and obese children varied from 3 to 87% (15, 16, 18, 21, 22, 25, 27, 28, 31, 38–56).

Even though the protecting factors keeping the children with MHO on the healthy side of the metabolic phenotype, despite the increase in adiposity, have not yet been fully elucidated, many authors have tried to identify the predictors of MHO status. The adiposity rebound, the early increase in BMI-SDS occurring in some children between the ages of 3 and 7 years, is known to be a significant risk factor for later obesity (57). A recent study assessing the prevalence of MHO among 31-year old adults from the Northern Finland Birth Cohort 1966 found that at the age of adiposity rebound, females with MHO were five and a half months younger and males with MHO were 4 months older, compared to the MUO group. Study findings demonstrated that the time of adiposity rebound might also have a role in defining the future cardiometabolic risk status in adulthood (58).

Pubertal status also plays a significant role in the development of both MHO and MUO phenotypes in youth with obesity. Puberty is a period of dynamic physiological changes, including a well-described physiological decrease in insulin sensitivity (59). A longitudinal study conducted with 2017 children with obesity over the course of 1 year showed that entering puberty doubled the risk for switching from MHO to the MUO phenotype, whereas changing from the mid to late pubertal stage nearly tripled the likelihood for crossing over from MUO to MHO, according to the results of multiple logistic regression analyses adjusted for age, sex, and changes of BMI-SDS (15). These findings highlight the importance of distinguishing physiological and pathological aspects of puberty within the MHO/MUO phenotypes in youth. Other studies have demonstrated that being in the earlier stages of pubertal development was a predictor for being metabolically healthy (15, 16). Diet and lifestyle habits may also contribute significantly to the MHO profile during puberty. A Canadian longitudinal study of 48 participants with MHO, who were followed up for 2 years as they entered puberty, showed that every additional daily portion of fruit and vegetables decreased the risk of converting to metabolically unhealthy obesity by 39% (60). The same study showed that more hours of screen time, and diet low in protein and high in saturated fat and sugar-sweetened beverages, were associated with the development of the MUO profile (60).

In an attempt to address the predictors of the MHO phenotype in youth, a recent large-scale, cross-sectional Chinese study of 7,926 children and adolescents aged 7–16 years in all weight ranges found that those with the metabolically healthy obesity phenotype were younger, and had lower BMI-SDS, WC, and body fat measurements (38). Another study conducted in Saudi Arabia with more than a thousand children with obesity also found that a younger age, lower BMI-SDS, and WC measurements, and female gender were predictors of MHO in children (41). A recent study conducted in Germany of a cohort of 246 children aged 7–18 years with overweight and obesity demonstrated that children with MHO were younger, had lower BMI-SDS and lower WC measurements, and lower plasma levels of uric acid and C-peptide, but were more likely to be male (42).

Among children and adolescents with overweight and obesity, the most consistent predictors for being metabolically healthy, as described in the previous studies were lower WC measurements (16, 18, 21, 22, 25, 28, 38, 41, 48, 49, 51, 55, 61, 62), lower BMI-SDS (22, 25, 38, 41, 42, 44, 48–50, 63), younger age (15, 16, 25, 38, 41, 42, 48, 50), and lower body fat measurements (21, 23, 25, 38, 44, 61–63). Nevertheless, the findings of several studies have also implicated additional potential predictors of MHO. Dietary patterns such as higher fruit and vegetable intake (60, 63), less protein intake (49), lower junk food consumption (50), lower fat intake (18), and lower frequency of soft drinks consumption (51) were associated with MHO phenotype. Likewise, lifestyle habits (e.g., less sedentary time) (27, 50, 64), higher levels of moderate and moderate-to-vigorous physical activity (18, 27, 49), and walking to school were also associated with a favorable metabolic profile (51). Sleep indicators have also been investigated and according to a study by Nasreddine et al., the weekly frequency of daytime napping was associated with the MHO profile (41). A South Korean study found that very short sleep duration (<5 h) when compared to a group with normal sleep duration (8–10 h), increased the risk of being metabolically unhealthy, independently of weight status (46).

Socio-economic features, such as having parents with a better socioeconomic status (51, 63) were found to be protective, in addition to higher birth weight (51), having generalized rather than central or combined obesity (65), lower waist-to-height ratio (25) and having a higher appendicular skeletal muscle mass in boys (66). The absence of *Acanthosis nigricans* (44, 48, 51) and laboratory markers of lower insulin resistance (16, 21, 28, 31, 44, 48, 61) as well as a lower degree of hepatic steatosis (22, 62) have also been investigated and shown to be predictors of MHO phenotype. In respect of other serum markers, decreased serum levels of ferritin and hemoglobin (67), osteonectin, retinol binding protein 4 (RBP-4), and leptin/adiponectin ratio (21, 26, 40) have been associated with the MHO phenotype.

## Mechanisms of Development of Metabolic Disturbances in Pediatric Obesity

Various hypotheses have been proposed to elucidate the underlying factors leading to the development of metabolic disturbances in pediatric obesity, and the mechanisms preserving the favorable MHO profile. The development of insulin resistance has been suggested as one of the main underlying etiological factors leading to metabolic disturbances in obesity (68, 69). Insulin affects the metabolic function of many organs including muscle tissue, adipose tissue, and the intestine, with a consequent multitude of metabolic disturbances arising with the development of insulin resistance (68). One of the most significant consequences of obesity is the development of impaired glucose tolerance (IGT) and type 2 diabetes (T2D). The transition from IGT to T2D in adults is estimated to occur within 5–10 years, although this process may be accelerated in some individuals (70, 71). Weiss et al. showed that weight gain in children was closely associated with deterioration of glucose regulation assessed using the oral glucose tolerance test (OGTT). They also found that an increased amount of adipose tissue, even if the BMI remained constant due to increase in height, was associated with a decrease in insulin sensitivity and progression to IGT from previously normal glucose regulation during OGTT (72). Findings from several studies have also confirmed that some children with obesity, and their relatives, display a low insulin secretion capacity to maintain euglycemia in insulin-resistant states (73–77). Therefore, a hereditary β-cell capacity to overcome significant insulin resistance may be considered as one of the potential mechanisms underlying completely normal glucose regulation in children with MHO.

Adipose tissue inflammation is considered to be one of the main factors underlying the development of insulin resistance in obesity (78–80). It is well-known that adipose tissue is a highly active endocrine and metabolic organ, sensing energy requirements, and secreting hormones (adipokines) and cytokines (anti-inflammatory and pro-inflammatory cytokines) (81). The balance between pro-inflammation and anti-inflammation shifts to the direction of inflammation due to an increase in the amount of adipose tissue accumulated in the adipocytes, although the exact mechanisms triggering inflammation are still being investigated (82). Increased concentrations of free fatty acids in adipocytes could activate endoplasmic reticulum and homeostatic stress and inflammation via the pro-inflammatory cytokines c-Jun N-terminal kinase (JNK) and nuclear factor-kappa B (NFκB) (81). Activation of these cytokines promotes monocyte chemoattractant protein-1 (MCP-1) response which leads to the migration of monocytes to the adipocyte tissue. MCP-1 is a key factor for monocyte migration to adipocytes, further cytokine release and the eventual development of atherosclerosis (83). Activation of the inflammatory cascade leads to the activation of serine kinases and the release of other molecules, such as galectin-3 and leukotriene B4 (LTB4), which directly decrease insulin sensitivity, thus resulting in insulin resistance (84, 85). There is also evidence suggesting that inflammation in obesity could be triggered by higher circulating levels of lipopolysaccharides (LPS), which are produced by gram negative bacteria in the intestine, in individuals with obesity. In obesity, increased intestinal permeability leads to the increased entry of LPS into the systemic circulation. These LPS are believed to trigger the inflammation process via activation of Toll-like receptor 4 (TLR4) (86). Studies have also shown the importance of CD4, CD8, T-helper 1, and T-helper 2 cells in the development of inflammation in obesity, with alterations in numbers of these immune cells influencing the inflammation status in both humans and mice (87–89).

The distribution of adipose tissue has a critical role in the metabolic health status. The lipids in the human body are stored mainly in the subcutaneous adipose tissue compartment. There is evidence that increased ratio of the abdominal subcutaneous adipose tissue to total body fat mass is associated with the metabolically unhealthy phenotype, while the increase in the gluteofemoral subcutaneous adipose tissue seems to be positively associated with MHO (90, 91). Additionally, when the storage capacity in the subcutaneous adipose tissue is exceeded, lipids are stored in the form of ectopic depositions in non-adipose tissue, such as muscle and liver, separate from the adipose tissue compartment. Several studies have shown that ectopic adipose tissue depositions in the liver, i.e., fatty liver, is significantly stronger predictor of MUO phenotype compared to the other ectopic fat deposition locations, which supports the important role of non-alcoholic fatty liver disease (NAFLD) in the development of the metabolically unhealthy phenotype (36, 92–96). However, in the obesity spectrum, there are individuals able to expand their subcutaneous adipose tissue without increasing the visceral fat content, which leads to preserved insulin sensitivity and the MHO phenotype (12).

Adipokines synthesized in adipocytes from different adipose tissues (e.g., adiponectin and leptin), may contribute to the metabolically healthy or unhealthy status (36, 97–99). Higher adiponectin levels have a protective influence and are associated with preserved insulin sensitivity in adolescents (40, 100). Several studies have shown an association of single nucleotide polymorphisms (SNPs) in the adiponectin gene with the development of T2D (101). Weight loss interventions and female gender have also been found to be related with high adipokine levels (102–104). However, increased levels of other adipokines, such as leptin, omentin, vaspin, resistin, and retinol binding protein 4 (RBP4) are associated with the development of obesity-related metabolic disturbances (105, 106). Adipokines synthesized from visceral fat, especially from intrahepatic adipose tissue, have been associated with the MUO phenotype (36, 97–99). Adipocyte hyperplasia and hypertrophy has also been associated with obesity-related metabolic disturbances (107). As a result of the increased fat deposition in adipocytes, various hormones and cytokines are secreted, causing recruitment of pre-adipocytes and adipocyte remodeling (108). Adipocyte remodeling occurs as a result of the harmonious interplay of many factors, with proliferator-activated receptor γ (PPARγ) playing the most important role (109). There is evidence that PPARγ could decrease the level of inflammation and insulin resistance, thus preventing metabolic deterioration (110). Moreover, the adipocyte hypertrophy and increased lipolysis lead to the leakage of free fatty acids, with smaller adipocytes having a higher lipogenesis/lipolysis ratio, which contributes to insulin resistance (111).

The liver plays a major role in both carbohydrate and lipid metabolism through several metabolic pathways, with the ectopic accumulation of lipids in the liver causing NAFLD and having the strongest effect on the metabolic profile (107, 112, 113). Genome wide studies have identified genes linked to the hepatic fat accumulation, also known as NAFLD genes (114). Similar to the adipose tissue, liver tissue also secretes inflammatory cells and cytokines (hepatokines) which contribute to the metabolically unfavorable profile in NAFLD (94, 115, 116). NAFLD is associated with the accumulation of ceramides and diacylglycerols, leading to insulin resistance via insulin receptors and signaling pathways (117). In the state of insulin resistance in NAFLD, insulin is unable to suppress the hepatic glucose production with the worsening of the glycemic control, and at the same time lipogenesis continues, further contributing to the atherogenic lipid profile in NAFLD with increased risk of cardiovascular complications (118–121).

Genetic factors also play a role in the development of MHO and MUO phenotypes in youth with obesity. Recently published genome wide association studies have shown the relationship between some genetic loci and BMI (122, 123), adipose tissue distribution (visceral or subcutaneous) (124), and leptin levels (125). The insulin receptor substrate 1 locus (IRS1) has been found to be associated with increased body adipose tissue content, but a decreased cardiometabolic risk, thus it is considered to be a gene associated with the MHO phenotype (126). On the other hand, loci such as fat mass- and obesity-associated (FTO) and near sprouty homolog 2 (SPRY2) have been found to be associated with a higher cardiometabolic risk (126). Similar to the IRS1 locus effects, other variants (COBLL1/GRB14) have also been found to be associated with MHO profile despite increased body fat percentage, possibly due to favorable fat distribution (subcutaneous vs. visceral), with other loci (PLA2G6, TOMM40) potentially associated with MHO phenotype being studied (126–128).

## Treatment and Outcomes of MHO in Childhood

If not diagnosed early, prevented and treated, obesity in children and adolescents leads to the development of T2D, hypertension, dyslipidemia, atherosclerosis, and consequently a higher incidence of cardiovascular premature death in adulthood (6, 129). Although this section aims to analyze screening and therapeutic strategies for youth with obesity in respect to their metabolically healthy/unhealthy status, it is important to be aware that obesity represents a continuum gradient of risk for cardiometabolic diseases, and that this young population may benefit from early prevention and treatment as long as it is a problem likely to persist in adulthood (13, 129).

Although children with MHO are usually defined as being without traditional cardiometabolic risk factors within the MS definition, several studies have detected the presence of other features among this population, such as: hepatic steatosis, increased carotid intima-media thickness, and inflammatory biomarkers, as well as higher degree of visceral fat accumulation, higher birth weight, adipose cell size, and different gene expression-encoding markers in adipose tissue (4, 8, 130–134). The diverse characteristics found in children with MHO may indicate they pursue a different metabolic phenotype compared to their peers of normal weight (7, 18). The data assessing NAFLD and its influence on the crossover from MHO to MUO, although with growing importance, are scarce in the pediatric age and yet inconclusive (4, 135, 136). Therefore, it is important to identify the group of children and adolescents with MHO who would benefit the most from early detection and intervention (13). Longitudinal studies have shown discrepancies in the results concerning the stability of the MHO/MUO status from childhood to the adulthood, depending largely on the length of assessment (15, 58). Thus, it is possible that previous cross-sectional studies and studies that did not take the pubertal stage into consideration have provided limited results (6). On the other hand, findings from long term studies have indicated that MHO status is more stable in adulthood (i.e., children and adolescents are more likely to remain MHO if they reach adulthood as MHO) (30). In the study by Li et al., these children with MHO did not have increased carotid-artery atherosclerosis compared with normal weight children, when assessed in adulthood (30). However, they presented with increased frequency of other cardiometabolic risk factors in adulthood, such as higher blood pressure levels and insulin resistance. These results suggest that all youth with obesity, including children and adolescents with MHO, might benefit from the early detection of obesity and weight-loss intervention programs (30).

The development or deterioration of cardiometabolic risk factors in children with MHO could potentially be prevented using specific measures such as low-intensity exercise. It is plausible that metabolic deterioration and crossover to MUO could be avoided in youth with MHO if a modest weight reduction is achieved or weight gain prevented (4). Although only studied in adults with MUO, besides the amount of weight loss, fatty liver at baseline was found to predict the response of conversion from MUO to a metabolically healthy phenotype (136, 137). Thus, by stratifying youth with obesity into groups at relatively lower and higher risk (MHO vs. MUO), the traditional one-size-fits-all treatment approach in managing obesity could be avoided. On the other hand, the continuum gradient of risk development for cardiometabolic diseases explains the importance of proper screening and an early start of treatment in both MHO and MUO children, although the metabolic profile of MHO individuals of pediatric age is almost indistinguishable from that of lean individuals (9). There is a possibility that MHO children may require less frequent follow-up or less intensive treatment, or possibly a different treatment approach compared to their MUO peers. However, this precondition needs to be evaluated in future studies, and in the meantime MHO children should be treated as all children with obesity, with special attention paid to the possibility of a crossover to MUO during puberty.

The management of children with MHO should not differ from the management of any child with obesity and therefore should emphasize preventive strategies, such as the promotion of a healthy lifestyle, especially targeting those with early onset obesity or rapid weight gain (60). In general, adults with obesity may respond differently to dietary and physical activity interventions for weight loss, and there is a hypothesis that MHO individuals may not metabolically benefit from these interventions (9). However, there are no findings to date, supporting this hypothesis in the population of youth with MHO. It has also been observed that children and adolescents are more likely to preserve their MHO status the less sedentary they are and the more time they spend in moderate-to-vigorous physical activities (27). Promoting a healthy diet should also be in the focus of preventive strategies. Although sometimes contradictory, several studies have shown that youth with MHO who ate fewer daily portions of fruits and vegetables, less meat, and had a higher intake of saturated fat were more likely to become MUO (18, 51, 60).

More studies are required to clarify the effects of different therapeutic approaches for the MHO profile in childhood. Most of the current data available is derived from studies performed in adults, and results from longitudinal studies of youth with obesity (both MHO and MUO) will hopefully help to identify appropriate intervention strategies for children with obesity, according to their metabolic health status. Promising research linking MHO patients and additional cardiometabolic risk factors such as NAFLD, inflammatory biomarkers and carotid intima-media thickness, although not currently included in either MS or MHO consensus criteria, will be useful to identify and manage both MHO and MUO alike.

## Conclusions

In conclusion, the use of a single/universal definition of MHO in childhood is highly beneficial for advancement in the field. We recommend use of the international consensus-based definition proposed by Damanhoury et al. to define the MHO phenotype in youth with obesity. When accessible, additional measures of “metabolic health” can also be included. This is crucial in order to have comparable results and further our understanding of the MHO phenomenon in children and adolescents. More data is needed in order to indentify the parameters which have the highest ability to predict clinically significant outcomes.

Future studies will improve our understanding of the prevalence, predictors and protective factors in MHO, as well as the underlying mechanisms leading to the crossover from MHO to MUO during puberty. The MHO status should be defined and controlled for during longitudinal interventional studies, in order to account for possible differences in treatment outcomes compared to the rest of the population with obesity. This could influence the development of targeted preventive and treatment strategies for youth with obesity with or without cardiometabolic risk factors in the future. Until then, the same therapeutic approaches and weight goals should be used in children with MHO, with the emphasis on the prevention and promotion of healthy lifestyle habits.

## Author Contributions

RV conceptualized the manuscript, drafted the sections regarding background, and definitions of MHO. MY drafted the section regarding prevalence and predictors. MA drafted the section regarding mechanisms. TD drafted the section regarding the treatment and outcomes. All authors reviewed and revised the manuscript and approved the final version.

### Conflict of Interest

The authors declare that the research was conducted in the absence of any commercial or financial relationships that could be construed as a potential conflict of interest.
